# Combined detection of serum CTX-II and COMP concentrations in osteoarthritis model rabbits: an effective technique for early diagnosis and estimation of disease severity

**DOI:** 10.1186/s13018-016-0483-x

**Published:** 2016-11-22

**Authors:** Bin Bai, Yanqin Li

**Affiliations:** 1 Orthopedics Department, The First Affiliated Hospital, Xi’an Jiaotong University, West YANTA Road 227#, Xi’an, Shaanxi 710061 China; 2 School of Public Health, Health Science Center of Xi’an Jiaotong University, Xi’an, Shaanxi 710061 China

**Keywords:** Osteoarthritis, Early diagnosis, CTX-II, COMP, Severity

## Abstract

**Background:**

Early diagnosis of osteoarthritis (OA) is difficult. Cartilage oligomeric matrix protein (COMP) and crosslinked C-telopeptides of type II collagen (CTX-II) are two markers which can potentially predict the destruction of articular cartilage of early OA. To comprehensively evaluate the diagnosis value of serum COMP and CTX-II markers in OA, the longitudinal and combined measurement of serum COMP and CTX-II were performed at different stages of pathological process of OA in adult rabbits with OA, compared with the sham-operated rabbits.

**Methods:**

Thirty-six adult white rabbits were randomly divided into two groups, the OA group and the control group (*n* = 18 per group). OA models were established by anterior cruciate ligament transection. Sham operations were performed in the control group. Before the surgery and at weeks 2, 4, 6, 8, 10, and 12 after surgery, serum CTX-II and COMP concentrations were detected using sandwich-ELISA in all rabbits. Three rabbits in each group were killed at weeks 2, 4, 6, 8, 10, and 12 after surgery, and femoral condyle specimens were collected. Histological changes in articular cartilage were evaluated according to the Mankin scoring criteria.

**Results:**

At each time point, the Mankin scores and serum concentrations of CTX-II and COMP were significantly higher in the OA group than in the control group. In addition, in the OA group, there was a significant relationship between the CTX-II and COMP concentrations and the Mankin score.

**Conclusions:**

Early dynamic combined detection of serum CTX-II and COMP concentrations is effective for early OA diagnosis and evaluation of OA severity.

## Background

Osteoarthritis (OA) is one of the most common bone and joint aging diseases; it can cause joint pain and disability [1]. Approximately 9.29% of the US population is diagnosed with symptomatic knee OA by 60 years of age [2]. However, the etiology and pathogenesis of OA are still unclear and it is still an incurable disease [3].

There are no sensitive diagnostic techniques other than classical radiography; accordingly, OA is often not detected until the middle or end stage [4, 5]. Owing to the absence of disease-modifying treatments, joint replacement is the only effective treatment for end-stage OA; other treatments cannot effectively alleviate pain or improve joint function [4–6]. The earlier OA is diagnosed and treated, the better the prognosis. Therefore, there is an urgent need to develop techniques that are more effective than radiography in clinical practice.

Some structural molecules and fragments derived from bone, articular cartilage, and the synovium, all of which are affected by OA, are candidate OA biomarkers [7–11]. Cartilage oligomeric matrix protein (COMP) and crosslinked C-telopeptides of type II collagen (CTX-II) are two biochemical markers; they are degradation products of joint tissues, especially the cartilage extracellular matrix, and can potentially predict the destruction of articular cartilage in OA [12–15]. However, many studies of early OA diagnosis have only measured one of these two biomarkers at a single time point during the pathological process of OA.

To evaluate the diagnostic value of serum COMP and CTX-II markers in OA comprehensively, a longitudinal analysis of the combined measurement of serum COMP and CTX-II was performed during the pathological process in adult rabbits with OA, using rabbits that received a sham operation as controls. In addition, the relationships between COMP and CTX-II concentrations and histological changes in articular cartilage were analyzed.

## Methods

### Experimental animals

Thirty-six healthy mature white rabbits (2.14–2.58 kg, 11–12 months old, 18 males and 18 females) were obtained from the Animal Experimental Center at Xi’an Jiaotong University. None of the rabbits was administered any medication or used in any previous experiment. The animals were acclimatized for at least 1 week before the start of the experiment. The rabbits were randomly divided into two groups, the control and OA groups (18 rabbits per group). Before the operation, 3% sodium pentobarbital (1 mL/kg) were injected intraperitoneally to the rabbits for anesthesia. All rabbits in the OA group underwent anterior cruciate ligament transection (ACLT) in their left hind knees under arthroscopy to induce OA [16]. The same surgical techniques except for ACLT were applied to the rabbits of the control group. After the operation, all rabbits in the two groups acted freely in cages, and their health status were observed twice daily. This study was approved by the Animal Care and Use Committee of Xi’an Jiaotong University.

### Serum collection and storage

Before surgery and at weeks 2, 4, 6, 8, 10, and 12 after surgery, 5 mL of blood was drawn from the ear margin vein of each rabbit in the control and OA groups. The blood was centrifuged for 15 min at 1000×*g* at room temperature. The supernatant was collected, divided, and stored at −80 °C until use (≤6 months). Blood collection tubes were disposable and non-pyrogenic and did not contain endotoxins.

### Tissue collection and histological analysis

At 2, 4, 6, 8, 10, and 12 weeks after surgery, three rabbits in each group were randomly selected and were administered euthanasia. The medial femoral condyles were cut from all rabbits and fixed in 10% buffered formalin for 72 h. Then the tissues were decalcified with 10% EDTA solution (pH = 7.4) on a 48-h/48-h/72-h cycle for approximately 10 weeks. After decalcification, the tissues were dehydrated in a graded series of ethanol and xylol and subsequently embedded in paraffin. For hematoxylin and eosin (HE) staining and toluidine O-staining, the paraffin tissue blocks were sectioned (5 μm). All sections were stained in a single batch. The histopathology changes in articular cartilage were assessed in stained sections according to the OA Histological Histochemical Grading System (HHGS) introduced by Mankin et al. [17]. A pathologist who did not know the grouping evaluated all the sections.

### Analysis of serum CTX-II and COMP concentrations

The serum CTX-II concentrations of animals in each group were also measured using the quantitative sandwich enzyme immunoassay technique (Rabbit CTX-II ELISA Kit, Cusabio Biotech Co., Ltd., College Park, MD, USA). Standard or sample (100 μL) was added to each well, and the plate was covered and incubated for 2 h at 37 °C. The liquid in each well was removed and 100 μL of anti-biotin antibody was added to each well. The plate was covered and incubated for 1 h at 37 °C. Then, each well was washed with Wash Buffer three times, 100 μL of HRP-avidin was added to each well, and the plate was covered and incubated for 1 h at 37 °C. Each well was washed with Wash Buffer five times, 90 μL of TMB substrate was added to each well, and the plate was incubated for 15–30 min at 37 °C. Subsequently, 50 mL of Stop Solution was added to each well. The optical density (OD) of each well was determined within 5 min using a microplate reader at 450 nm. The CTX-II concentration was calculated by comparing the OD of each sample to the standard curve.

The serum COMP concentration of each group was measured using the sandwich enzyme-linked immunosorbent assay (Rabbit COMP ELISA Kit, Elab science Biotechnology Co., Ltd., Wuhan, China). Standard or sample (100 μL) was added to each well, and the plate was covered and incubated for 90 min at 37 °C. Then, the liquid in each well was removed and 100 μL of Biotinylated Detection Ab Working Solution was added to each well. The plate was covered and incubated for 1 h at 37 °C. Then, each well was washed with Wash Buffer three times, 100 μL of HRP-conjugate working solution was added to each well, and the plate was covered and incubated for 30 min at 37 °C. Each well was washed with Wash Buffer five times, 90 μL of Substrate Solution was added to each well, and the plate was covered and incubated for 15 min at 37 °C. Then, 50 μL of Stop Solution was added to each well. The OD value of each well was determined immediately using a microplate reader at a wavelength of 450 nm. The COMP concentration was calculated using the standard curve.

### Statistical analysis

The data are expressed as means ± standard deviation (SD). All statistical analyses were implemented in SPSS 13.0. The Mann–Whitney test was utilized to examine differences in the Mankin scores and the serum concentrations of COMP and CTX-II between the control and OA groups. Correlations were analyzed using Spearman’s rank correlation analyses. A *p* value of less than 0.05 was considered statistically significant.

## Results

### Histology

**Fig. 1:**
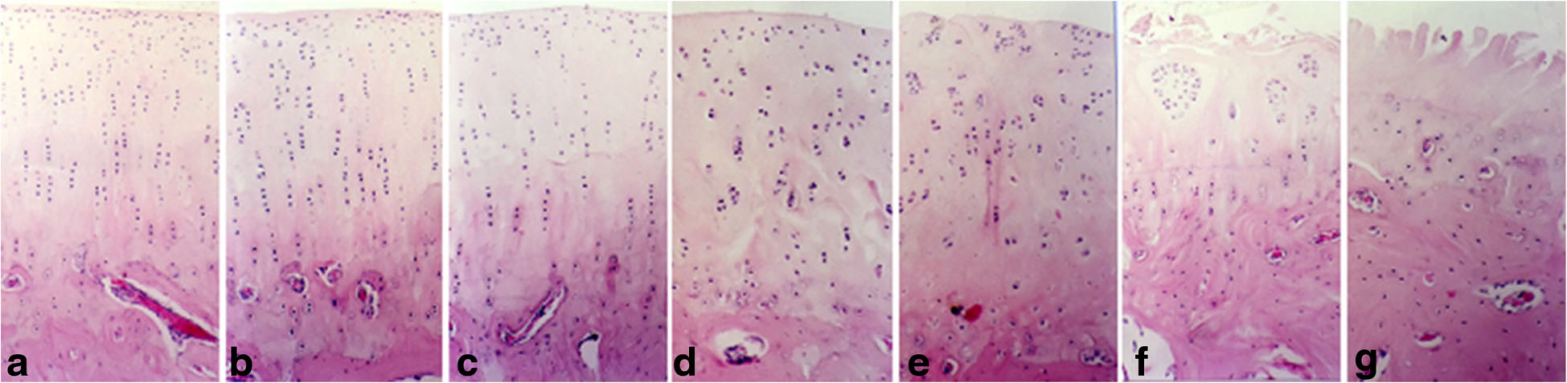
Hematoxylin and eosin (HE) staining of articular cartilage of rabbits in two groups (magnification: ×10). **a** Control. **b** Week 2 after surgery. **c** Week 4 after surgery. **d** Week 6 after surgery. **e** Week 8 after surgery. **f** Week 10 after surgery. **g** Week 12 after surgery

**Fig. 2:**
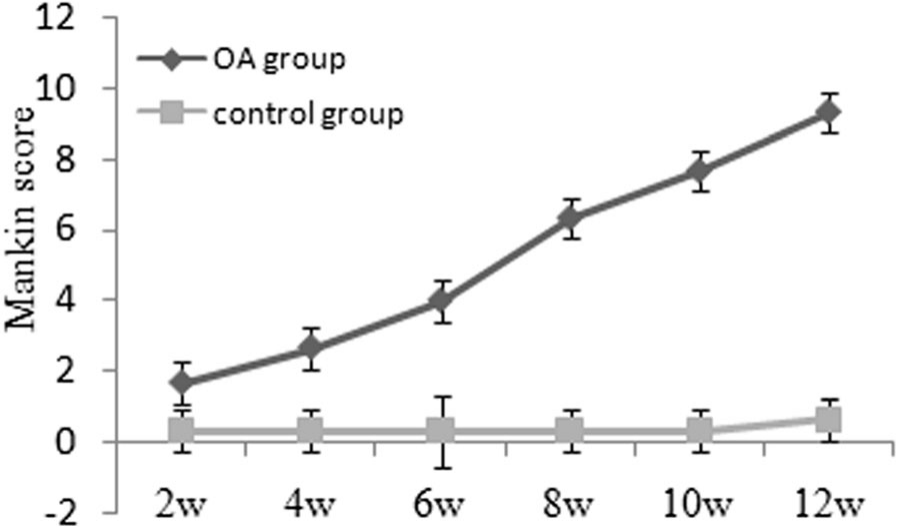
Mankin score of articular cartilage of rabbits in the OA and control groups at various time points

In the control group, the slices of HE staining exhibited a smooth and intact surface of articular cartilage. In addition, the chondrocytes in the middle zone were organized and the staining of matrix was homogeneous (Fig. 1a). However, in the OA group, the different manifestations of articular cartilage were observed at various stages. At week 2 after surgery, the articular cartilage surface showed also smooth and intact. However, the chondrocyte in the superficial zone distributed unequally (Fig. 1b). At week 4 after surgery, the articular cartilage surface became unsmooth and the chondrocytes in the middle zone were disorganized (Fig. 1c). At week 6 after surgery, the articular surface showed completely rough, and the chondrocytes in the middle and deep zones were completely disorganized (Fig. 1d). At week 8 after surgery, proliferative chondrocyte clusters emerged in the superficial zone (Fig. 1e). At week 10 after surgery, proliferative chondrocyte clusters became noted in the superficial and middle zones. The matrix showed a loss of staining (Fig. 1f). At week 12 after surgery, vertical clefts in the calcified zone were found in some specimens. Cell loss was noted in the middle and deep zones (Fig. 1g). The Mankin scores are shown in Fig. 2. Within each time point, the Mankin scores in the OA group were significantly higher than those in the control group were (*P* < 0.05). The Mankin scores of the OA group increased as OA progressed.

### CTX-II and COMP concentrations

**Fig. 3:**
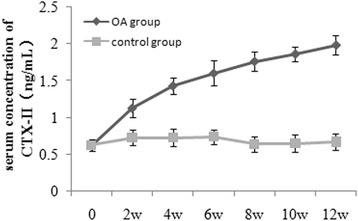
Serum CTX-II concentration in the OA and control groups at various time points

**Fig. 4:**
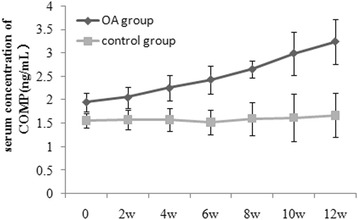
Serum COMP concentration in the OA and control groups at various time points

**Fig. 5:**
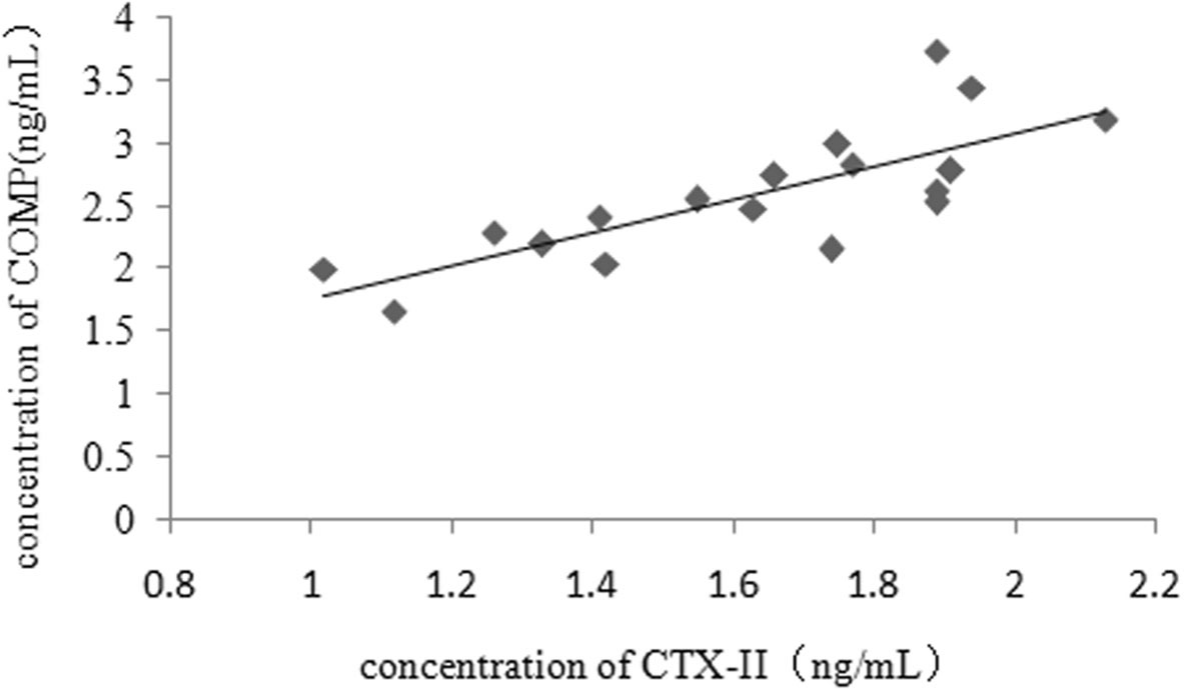
Correlation between the serum concentrations of COMP and CTX-II

The concentration of CTX-II in rabbits of the control and OA groups at various time points are summarized in Fig. 3. The CTX-II concentration in the OA group was higher than that in the control group (*P* < 0.05). Similarly, the COMP concentrations of rabbits in the control and OA groups at various time points are summarized in Fig. 4. The COMP concentration was higher in the OA group than in the control group (*P* < 0.05). In addition, the concentrations of serum CTX-II and COMP increased in the OA group with the progression of OA. There was a positive correlation between the serum CTX-II concentration and serum COMP concentration (*r* = 0.823, *P* < 0.001) (Fig. 5).

### Correlation between the serum concentrations of COMP and CTX-II and the Mankin score of the corresponding articular cartilage

**Fig. 6:**
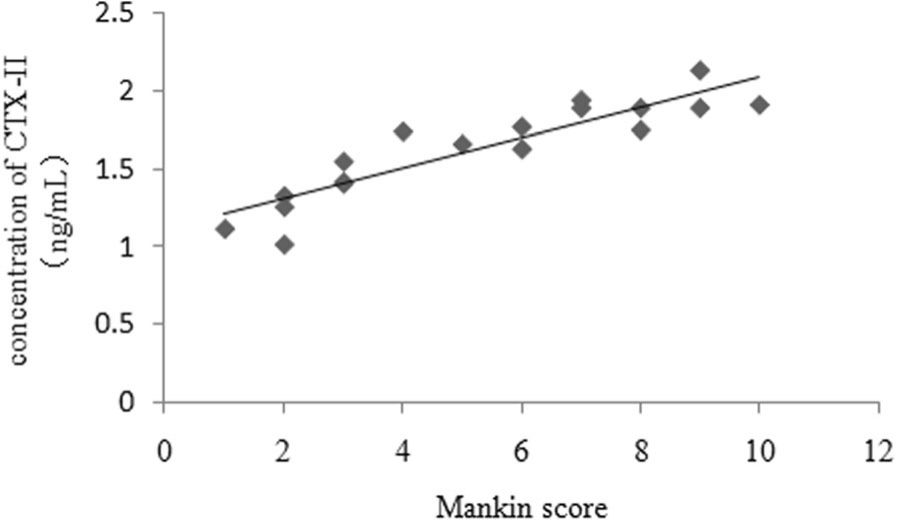
Correlation between the serum concentration of CTX-II and the Mankin score of the corresponding articular cartilage

**Fig. 7:**
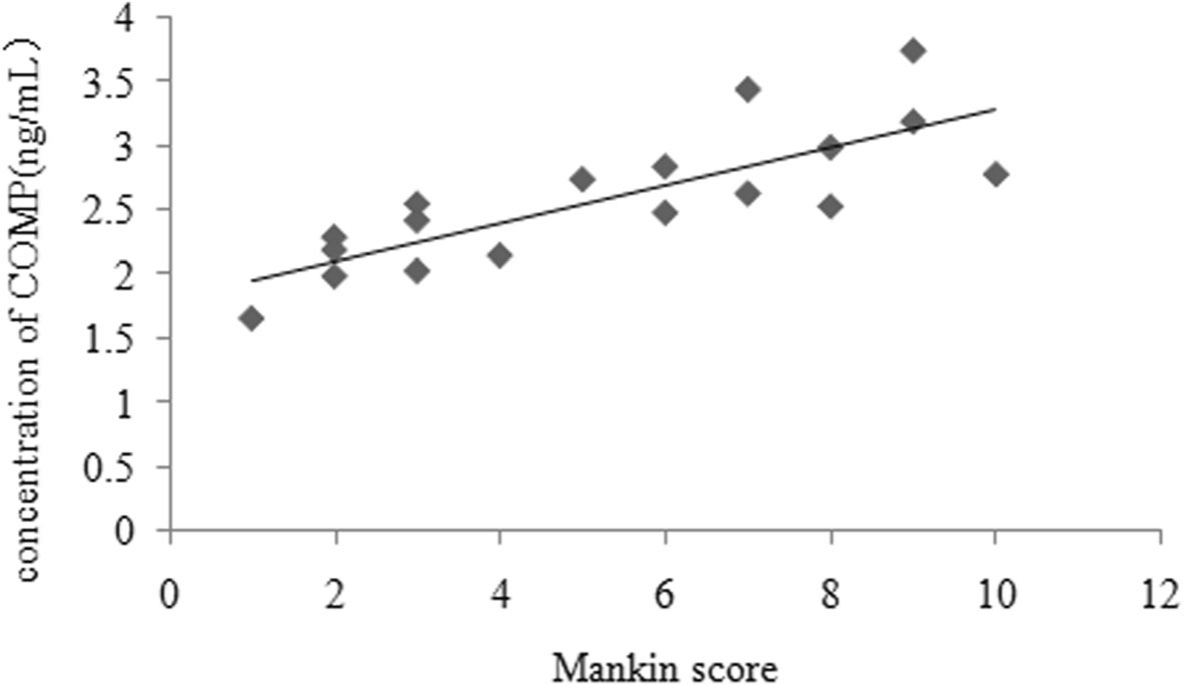
Correlation between the serum concentration of COMP and the Mankin score of the corresponding articular cartilage

There was a positive correlation between the serum concentration of CTX-II and the Mankin score of the corresponding articular cartilage (*r* = 0.882, *P* < 0.001) (Fig. 6). Similarly, there was a positive relationship between the serum concentration of COMP and the Mankin score of the corresponding articular cartilage (*r* = 0.798, *P* < 0.001) (Fig. 7).

## Discussion

In this study, our main aim was to investigate the diagnostic value of CTX-II and COMP at the early stage of OA. The degeneration of articular cartilage, including the degeneration of cartilage cells and matrix, is the main pathological characteristic of OA [18]. However, matrix degeneration mainly results in the losses of proteoglycans and type II collagen. CTX-II is one of the main products of type II collagen degeneration. When type II collagen is degraded, CTX-II is released into the synovial fluid and absorbed by the serum. COMP, a tissue-specific protein, binds to type II collagen fibers and can stabilize the collagen fiber network of articular cartilage. When the articular cartilage is destroyed, COMP is released into the synovial fluid and absorbed by the serum. Accordingly, the serum levels of CTX-II and COMP reflect the metabolism of type II collagen fibers.

Tang et al. [19] found that the serum levels of CTX-II were significantly higher in patients with Kashin–Beck disease (KBD) or OA than in healthy adults. In our study, the serum concentration of CTX-II was higher in the OA group than in the control group at all time points after surgery. We found that the serum concentration of CTX-II increased beginning as early as week 2 after surgery. The same results have been obtained in rabbits with OA induced by ACLT in a previous study [12]. These results suggest that type II collagen fibers are destroyed at a very early stage of OA development. This inference is supported by our histological results, which indicated that there was minor damage in the articular cartilage of the OA group at week 2 after surgery. We also found that the serum concentration of CTX-II increased over time in the OA group. The same results have been obtained in previous studies [12, 20]. These results indicate that articular cartilage damage increased over time in the OA group. We also found that the increase in the CTX-II concentration was gradual, and the apparent peak was not detected at 12 weeks. However, an obvious peak was observed during the first 3 weeks following surgery in the study of Duclos et al. [20]. They inferred that the joint response was associated with postoperative inflammation owing to chirurgical alterations, which could influence cartilage collagen turnover. In this study, the operation was performed under arthroscopy, with slight surgical injury to the joint such that the impact to the balance structure of the joint was negligible. Therefore, the joint response was mild and no obvious peak was detected.

To investigate the correlation between the CTX-II concentration and the severity of articular cartilage degeneration, histological changes in the articular cartilage at various time points were evaluated based on the Mankin scoring system. We found that within each time point, the Mankin scores of the corresponding articular cartilage in the OA group were significantly higher than those in the control group. It indicated that the OA model induced in this study was effective. In this OA model, articular cartilage degeneration was a slow and gradual process, in accord with real histological processes of OA. We also found that the serum CTX-II concentration was positively correlated with the Mankin score of the corresponding articular cartilage in the OA group during the 12-week study. Higher serum CTX-II concentrations were associated with higher articular cartilage Mankin scores. The higher serum CTX-II concentration illustrated that more type II collagen was catabolized. The higher Mankin score indicated that the degeneration of articular cartilage was more severe. Tanishi et al. [21] found that individuals over 60 years old with OA grade 3 or 4 according to X-rays had significantly higher urinary CTX-II values than those with grade 0, 1, or 2. Jordan et al. [22] found that urinary CTX-II was strongly associated with the presence and severity of radiographic knee OA in men aged 59–70 years. Accordingly, the dynamic detection of the serum CTX-II concentration could reflect the severity of OA articular cartilage lesions and facilitate the early diagnosis of OA and predictions regarding the effect of clinical treatment.

Similarly, Fernandes et al. [23] found that patients with symptomatic knee OA present significantly higher serum COMP levels compared to healthy controls and those with non-symptomatic narrowings of the articular space. El-Arman et al. [24] reported that the serum and synovial fluid COMP levels are elevated and positively correlated with radiological joint damage in OA. Furthermore, Verma et al. [14] performed a case-control study to estimate the association of COMP with knee OA and found that the median (range) serum COMP levels are higher in OA patients than in control subjects. In our study, the serum COMP concentration of the OA group was also elevated compared with that of the control group at week 2 after surgery. At other time points, the serum COMP concentrations were also higher in the OA group than in the control group. Similar results were reported by Zuo et al. [12]. These results illustrated that the serum COMP concentration in the OA group was elevated at the early stage of OA and increased during the progression of OA. We also found that the increase in the serum COMP concentration was gradual in the OA group, and no peak was detected during the 12-week study.

We also investigated the correlation between the serum COMP concentration and the severity of articular cartilage degeneration. We detected a positive correlation between the serum COMP concentration and the Mankin score of the corresponding articular cartilage in the OA group during the 12-week study. Specifically, higher serum COMP concentrations were associated with higher Mankin scores for the corresponding articular cartilage. Higher serum concentration of CTX-II indicated that more type II collagen was metabolized. Lai et al. [25] reported that the serum COMP fragment concentration is highly correlated with the severity of OA and the progression of surgically induced OA in murine models. Clark et al. [15] demonstrated that in a population-based sample, serum COMP concentrations could distinguish an OA-affected subgroup from an unaffected subgroup and could reflect disease severity and multiple joint involvement in OA. Based on previous results and those of this study, the early dynamic detection of serum COMP concentrations could be used to monitor OA severity and to help diagnose OA at an early stage.

As biomarkers, the CTX-II and COMP concentrations could reflect the degree of type II collagen degeneration. However, the results of in vitro and ex vivo human cartilage experiments suggested that CTX-II is released by different enzymatic pathways; accordingly, CTX-II alone only partially reflects overall cartilage collagen degradation [13]. Furthermore, Dodge et al. [26] demonstrated that a small amount of COMP is produced by several mesenchymal cells, including synoviocytes and dermal fibroblasts. We found that the serum concentrations of CTX-II and COMP were positively correlated during the 12-week study. Therefore, the combined analysis of serum CTX-II and COMP concentrations could provide complementary information on disease development in OA and could be used to more accurately judge the severity of OA and diagnose it at an early stage.

This was one of few studies evaluating the value of CTX-II and COMP as biomarkers for the early diagnosis of OA and estimations of disease severity. This study had some limitations. The 12-week longitudinal study was not sufficiently long; accordingly, changes in CTX-II and COMP at the end stage of the disease were not observed. We only performed a histological analysis of medial femoral condyles, not including the lateral femoral condyle and tibial plateau. However, the results are reliable because some scientific and effective measures were taken in this study, such as an appropriate sample size, random grouping, sham-operation control, repeating, and blinding.

## Conclusions

The serum concentrations of CTX-II and COMP were significantly higher in the OA group than in the control group. The serum CTX-II and COMP concentrations increased as OA progressed. There was a positive relationship between the serum CTX-II and COMP concentrations and the Mankin score of the corresponding articular cartilage during OA progression. The serum concentrations of CTX-II and COMP were positively correlated. The dynamic combined detection of serum CTX-II and COMP concentrations has important applications for the early diagnosis of OA and the determination of OA severity.

## Abbreviations

COMP: Cartilage oligomeric matrix protein
CTX-II: Crosslinked C-telopeptides of type II collagen
OA: Osteoarthritis
